# Magnitude of iodine deficiency disorder and associated factors in Dawro zone, Southwest Ethiopia; the hidden hunger: a cross-sectional study

**DOI:** 10.1186/s40795-020-00345-8

**Published:** 2020-06-08

**Authors:** Agize Asfaw, Tefera Belachew

**Affiliations:** 1 Department of Public Health, College of Medicine and Health Sciences, Wolkite University, P.O.Box: 07, Gubre, Ethiopia; 2 Department of Nutrition and Dietetics, College of Public Health and Medical Sciences, Jimma University, P.O.Box: 378, Jimma, Ethiopia

**Keywords:** Iodine deficiency disorder, Urinary iodine excretion, Goiter, Dawro, Ethiopia

## Abstract

**Background:**

Iodine deficiency disorder (IDD) is continued to be a major public health problem in Ethiopia. The problem is worse but the data is scarce in some pocket areas of the country. This study was aimed to assess the magnitude of IDD and associated factors in southwest Ethiopia by using different biochemical and clinical indicators.

**Methods:**

Both community and school based cross- sectional study was conducted on school age children (6–12 years) from March 1 to 24, 2017. Simple random and systematic sampling techniques were applied to select districts and eligible children respectively. Household data was collected from children’s primary care takers by using structured questionnaire. A trained surgeon examined all children (*n* = 652) for goiter. Urine sample was collected from 20% of children (*n* = 130).

**Results:**

A total of 652 school children were participated in the study. Total goiter rate and median urinary iodine concentration (UIC) was 54.8% and 96.12 μg/l respectively. In multivariable logistic regression analyses student’s age was significantly (*P* < 0 05) associated with both goiter and median UIC. However; sex of students and family income were significantly (*P* < 0.001) associated with goiter and cassava consumption was significantly (P < 0.001) associated with UIC.

**Conclusion:**

As indicated by the median UIC, there was mild iodine deficiency in this study community. However; chronic iodine deficiency was still a severe public health problem (high goiter prevalence). In addition, there was low concentration of iodine in the salt and increased cassava cultivation and consumption indicating the potential risk in the area. Therefore, it is necessary to intensify IDD elimination activities along with teaching communities on techniques of cassava processing for household consumption.

**Trial registration:**

PACTR201809544276357. Retrospectively registered on 14, Sept 2018.

## Background

Iodine is a trace element sparsely distributed over the surface of the earth. It is essential for the synthesis of the thyroid hormones, thyroxin (T_4_) and triiodothyronine (T_3_), which are necessary for human growth and development. According to world health organization (WHO), United Nations children’s fund (UNICEF) and iodine global network (IGN), the recommended daily intake of iodine is 120 μg/day for school children (6-12 years) and 150 μg/day for adults. This means, on average the total quantity of iodine needed for an individual over a lifetime is only about one tea spoonful [1, 2].

Failure to have such small but very essential element can lead to several important consequences collectively known as iodine deficiency disorders (IDD). The spectrum of IDD includes impaired mental function, delayed physical development, goiter, hypothyroidism, cretinism, reproductive failure and increased infant mortality [3].

Among micronutrient deficiencies, iodine deficiency (ID) deserves highest priority, because it is the world’s single most significant cause of preventable brain damage and mental retardation in children [4, 5]. Despite the availability of a safe and simple solution for its elimination, iodized salt, it continued to be one of the main public health challenges in the world. The problem is worse in developing countries especially among population living in highland areas [6–8]**.**

### Factors associated with iodine deficiency

Even though, the main reason for IDD is a low dietary intake of iodine [9], several other factors also play significant role. Living in highland areas where the soil has low iodine content as a result of loss of iodine due to repeated leaching effects of snow, water, over-grazing by livestock, tree-cutting for firewood, and heavy rainfall, consumption of goitrogenic foods like cassava, millet, soya bean, kale, cabbage which interferes with the metabolism of iodine and other nutritional deficiencies including vitamin A, protein energy malnutrition and iron deficiency anemia, all contribute to the occurrence of IDD [10, 11].

Generally, factors that contribute to the occurrence of IDD are complex including environmental, social, economic, educational and service related as indicated by previous studies [12–14].

### Global iodine nutrition status and rationale for this study

Global studies conducted to assess iodine nutrition status over the past several decades had shown improved iodine nutrition status. According to these studies, in 1993 about 113 countries were iodine deficient. In 2003, after a decade, the number of iodine deficient countries declined to 54 [15]. After another decade, in 2013, this number decreased to 30 and currently in 2017 only 19 countries remain iodine-deficient [16, 17]. However; there was a sharp regional and urban/rural difference which still needs great global effort to combat IDD. There are great differences even in countries located within the same continent [18, 19].

Ethiopia is known for its mountainous topography and at risk of IDD. As indicated by national community based survey of 2007, the weighted total goitre prevalence was 35.8% and the median UIC level was less than 100 μg/l in all regions of the country [20]. Even though, IGN by its global scorecard of iodine nutrition of 2017 categorized the country as “adequate” in iodine intake at national level [21], several recently conducted sub-national studies revealed that the country is iodine deficient [11, 22–26]. In addition the availability of adequately iodized salt at household level was low [27].

In the current study area there is no data about the iodine status of the population, but there are several indicators for the existence of risk of IDD (the mountainous nature, high rain fall for most seasons of the year and availability and consumption of goitrogenic foods). Therefore, the major objective of this study was to assess the magnitude and predictors of IDD in Dawro zone, southwest Ethiopia and provide data for evidence based public health intervention.

## Methods

### Description of the study area

This study was conducted in Dawro zone, southwest Ethiopia. The zone is one of the 14 zones found in south nation nationalities and people’s region (SNNPR) with a total land area of about 4437 km^2^. The capital of the zone is Tarcha, which is found 500Km from Addis Ababa, the national capital in the south west direction. The total population of the zone was 702,517 in 2016 of which 50.9% are males and the remaining 49.1% are females. It has 3 different agro-ecological zones; high land with altitude over 2300masl (29%), middle land between 1500 to 2300 masl (41%) and low land below 1500 masl (30%). The annual temperature and rainfall ranges from 15.1 - 370c and 1800 – 2500 mm respectively [28, 29].

### Study design

Both community and school based cross- sectional study design was conducted from March 1 to 24, 2017. The study focused on school age children (SAC) aged 6–12 years. According to WHO/ UNICEF/IGN, these groups are a useful target for IDD surveillance because of their combined high vulnerability, easy access, applicability to a variety of surveillance activities and relative representativeness of the study population [2, 30].

### Source and study population

The source populations were all primary school children (6–12 years) in Dawro zone and the study populations were the same children from selected primary schools.

### Eligibility criteria

#### Inclusion

SAC (6–12 years) of both sexes from selected schools.

#### Exclusion

Children whose parents/guardians refuse to give consent and those who are outside the age range were excluded.

### Sampling technique

Two out of the five districts were selected by simple random sampling (SRS) and the town administration was included. Each district was stratified in to two agro-ecological zones based on their altitudes, highland (> 1500 m above sea level) and lowland (< 1500 m above sea level). For the town administration no need of stratification as it is the known low land. The list of primary schools (sampling frame) was obtained from district education offices and the town administration bureau. Again SRS was applied to select a primary school from each stratum and the town. Then, sample was allocated proportional to the number of students of each school. Eligible children in these schools were randomly selected and given an ID number. Finally, the following activities were carried out with the children and their mother/primary care taker:

A. **Clinical examination for goiter:** A surgeon from Tarcha general hospital trained for 3 days on thyroid gland examination and grading. The training was given by another surgeon working in department of surgery with special training on endocrinology. The trained surgeon examined all selected children by inspection and palpation. The goiter grading was performed by using WHO classification [2] as:

- Grade 0- Neither visible nor palpable in normal position (no neck swelling or normal)
- Grade 1 - Thyroid swelling which is not seen but palpable in normal position
- Grade 2 - Thyroid swelling which is both visible and palpable in normal position

B. **Urine Samples:** Casual urine samples of about 5 ml [30] was collected from 20% (*n* = 130) of children by using labeled plastic bottles with screw cap and put in an ice-packed cool box. The collected urine was transported to Tarcha general hospital laboratory for storage in a refrigerator at -4oc until it was transported to Ethiopian public health institute for analysis using modified Sandell-Kolthoff reaction. The result of UIC was expressed as micrograms of iodine per 100 ml of urine. Finally, the iodine status of children was classified by using WHO/UNICEF/IGN recommended cut-off points for UIC [2, 31, 32].
C. **Salt samples:** A sub-sample of 230 (^~^ 35%) households were selected from the main sample of 652. During data collection, interviewers asked every third households to provide two teaspoon of salt used for cooking. The collected sample was labeled with a code and submitted to principal investigator (PI). Then the sample was tested by trained laboratory technologist using iodine rapid test kit (MBI Kits International) for its iodine content. The iodine concentration was recorded as 0, < 15, or ≥ 15 PPM [2, 31, 32].
D. **Household interview:** Data collectors conducted household interview with the children’s mother or primary care taker by using pre-tested structured questionnaire. The questionnaire included socio-demographic, behavioral, nutritional and other factors that can possibly be associated with IDD.

### Operational definitions

- **Goiter Prevalence**:- Grade 1 and Grade 2 goiter collectively
- **Iodine content of salt samples:** 0 ppm (no iodine in the salt), < 15 ppm (inadequately iodized salt) and > 15 ppm (adequately iodized salt).

### Data processing and analysis

Data was re-checked for completeness and consistency after fieldwork. Then, coded on pre-arranged coding sheet by the PI and doubly entered into Epi Info version 3.5.3 and transferred to SPSS version 21 software packages. Before analysis it was cleaned and checked for consistency and possible outliers. Any incomplete or invalid data, outliers or missing values were evaluated against hard copy. Tables, graphs and figures were used to show frequencies and main findings.

A binary logistic regression model was fitted to identify factors associated with UIC and goiter. Variables with a *p*-value ≤0.2 in the bivariable analysis and those which frequently showed significant association with UIC and goiter in the previous studies were fitted into the multivariable logistic regression analysis and backward LR method was employed to control confounding. Both Crude Odds Ratio (COR) and Adjusted Odds Ratio (AOR) with the corresponding 95% Confidence Interval (CI) were calculated to show the strength of association. Variables with a *p*-value of ≤0.05 were considered as statistically significant.

### Data quality assurance

To ensure data quality, structured questionnaire was developed first in English then translated to Amharic by language expert and back to English to maintain its consistency. Five percent of it was pre-tested. Data collectors were trained for 3 days. Data was checked and cleared every day for completeness and consistency during the fieldwork. Close monitoring and supervision of data collectors was carried out to maintain data quality. Goiter examination was carried out by experienced surgeon from Gessa hospital. Twenty children (3% of the sample) randomly selected from the children examined for goiter and taken to Tarcha general hospital surgical department for re-examination by senior surgeon who was blinded about the result of the previous surgeon. When the result of both surgeons cross-checked, there was no misdiagnosis, but only 5 % misclassification was observed. That is one child, among the 20, was classified as having ‘grade 1 goiter’ by first surgeon but the second surgeon classified him as having ‘grade 2 goiter’.

## Results

### **Socio-demographic characteristics** of children

A total of 652 SAC (6–12 years) from six primary schools were included in the study. Two hundred ninety seven (45.6%) were male and 355 (54.4%) were female. The mean age ± SD of participant children was 9.24 ± 1.29 years.

### Goiter prevalence in children

All 652 SAC were examined for goiter. The goiter prevalence (Grade 1 + Grade 2) was 54.6%, of which 278 (78.1%) was grade 1 and the rest 78 (21.9%) was grade 2 goiter. Grade 0 means there is no visible or palpable swelling on the neck (normal). As indicated in Table 4, the prevalence of goiter increased with increasing age and feminine sex.

### Urinary iodine concentration in children

**Fig. 1:**
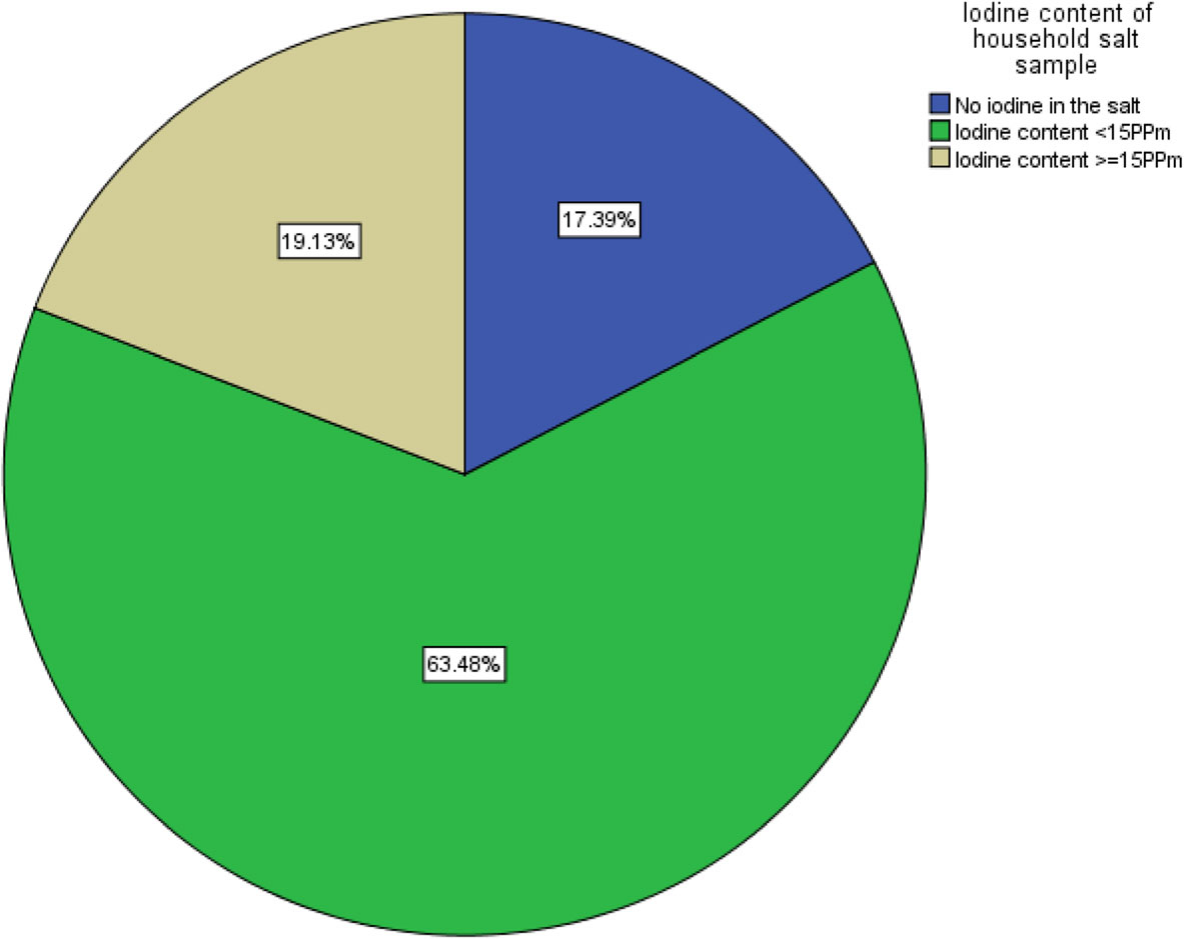
The iodine content of household salt sample, Dawro zone, southwest Ethiopia, 2017

The median UIC was 96.12 μg /l which is close to the WHO recommended cut-off 100 μg /l. When we observe the UIC values for the individual children, 66 (50.8%) of the children had UIC value < 100 μg/l (Fig. 1).

### Socio-demographic and economic **characteristics of parents**

The majority 642 (98.5%) of the respondents were female of reproductive age group. The mean age ± SD of participants was 29.9 ± 8.0 years and ranged from 15 to 49 years. Four hundred and sixteen (63.8%) of households had family size ≤5 and the average was 4.92 with SD of 1.92. More than half of the households (52.6%) gain less than 500 Ethiopian birr per month. Almost all participants 639 (98.0%) were Dawro by ethnicity, majority 489 (75.0%) were protestant followed by Orthodox 121 (18.6%) Christians. Most of them were married 522 (80.1%) and housewives in occupation 463 (71.0%). Three hundred eighty five (59%) had no formal education, whereas only 47 (7.2%) had college and above level in education (Table 1).

**Table 1:** Socio-demographic and economic characteristics of the study participants in Dawro zone, southwest Ethiopia, 2017, (*n* = 652)

Character	Frequency	Percent
Family Size		
< 5	416	63.8
> 5	236	36.2
Age of the responded parent		
15–24	162	24.8
25–34	311	47.7
35–44	127	19.5
45+	52	8.0
Age of the children		
7–9	356	54.6
10–12	296	45.4
Sex of the responded parent		
Female	642	98.5
Male	10	1.5
Sex of the children		
Female	355	54.4
Male	297	45.6
Ethnicity		
Dawro	639	98.0
Others^a^	13	2.0
Educational status of the responded parent		
Can’t read and write	287	44.0
Can read and write	98	15.0
Grade 1–8	129	19.8
Grade 9–12	91	14.0
College and above	47	7.2
Occupation		
House wife	463	71.0
Others^c^	189	29.0
Marital status of the responded parent		
Single^b^	130	19.9
Married	522	80.1
Religion of the responded parent		
Orthodox	121	18.6
Protestant	489	75.0
Catholic	35	5.4
Muslim	7	1.1
Geographic location		
Highland (Dega)	326	50.0
Lowland (Kolla)	326	50.0
Monthly family income (in Eth.Birr)		
< 500	343	52.6
500–1500	235	36.0
> 1500	74	11.4

^a^Konta, Wolaita, Amhara ^b^Divorced, widowed, separately living ^c^Farmer, Merchant, Government employee, Student and Daily laborer

### Iodine content of salt samples

The iodine content of salt sample (*n* = 230) was assessed at household level. According to the result, almost two-third, 146 (63.5%) of households were using inadequately iodized salt (< 15 ppm), 40 (17.4%) were using salt that had no iodine at all (0 ppm) and only 44 (19.1%) of the households were using adequately iodized salt (> 15 ppm).

### Use of Goitrogenic food

The result from household questionnaire had shown that goitrogenic foods like Cassava, Cabbage, Kale, Sorghum, were consumed with different frequency in a week. On the other hand consumption of iodine sources, especially sea foods like fish were scarce, but staples such as egg, milk and milk products were common (Table 2).

**Table 2:** Selected types of food used by the respondent and her/his family with its frequency of consumption per week in Dawro zone, southwest Ethiopia, 2017, (*n* = 652)

Food type	Frequency of consumption per week				
	Every day No (%)	4-6x/week No (%)	1-3x/week No (%)	Sometimes No (%)	Never No (%)
**Cassava**	**0 (0.0)**	**17 (2.6)**	**114 (17.5)**	**232(35.6)**	**289 (44.3)**
**Cabbage**	**0 (0.0)**	**33 (5.1)**	**336 (51.5)**	**222 (34.0)**	**61 (9.4)**
**Kale**	**155(23.8)**	**205 (31.4)**	**250 (38.3)**	**33 (5.1)**	**9 (1.4)**
**Sorghum**	**3 (0.5)**	**62 (9.5)**	**162 (24.8)**	**228 (35.0)**	**197 (30.2)**
**Sweet potato**	**26 (4.0)**	**79 (12.1)**	**217 (33.3)**	**243 (37.3)**	**87 (13.3)**
**Soya bean**	**0 (0.0)**	**4 (0.6)**	**100 (15.3)**	**176 (27.0)**	**372 (57.1)**
Fish	0 (0.0)	0 (0.0)	49 (7.5)	60 (9.2)	543 (83.3)
Egg	5 (0.8)	34 (5.2)	301 (46.2)	287 (44.0)	25 (3.8)
Milk & milk products	33 (5.1)	55 (8.4)	327 (50.2)	226 (34.7)	11 (1.7)

Bolded food items considered as containing goitrogenic substances (risk)

Non-bolded food items considered as sources of iodine (Protective)

### Prevalence of IDD in the study area

According to WHO, UNICEF and IGN, the presence and severity of ID in the general population can be indicated by its appearance among representative sample of SAC. In this study, the median UIC in SAC was 96.12 μg/l, which is close to the WHO recommended cut-off 100 μg/l reflecting mild iodine deficiency in this study population.

### Factors associated with urinary iodine concentration

In the bivariate logistic regression analysis, age and sex of children, family size and cassava consumption were significantly associated with UIC level. Then all variables with *P* < 0.20 and those which frequently showed significant association with UIC in the previous studies [11, 13, 19, 24] were entered in to multivariable logistic regression analyses. Student’s age, family size, geographic location and cassava consumption were significantly associated with UIC (Table 3).

**Table 3:** Factors associated with UIC level at primary school children in Dawro zone, southwest Ethiopia, 2017, (*n* = 130)

Variables with Category	UIC (μg /l)		Crude OR (95% CI)	Adjusted OR (95% CI)
	Sufficient^b^	Insufficient^c^		
**Age of the student**				
7–9 years	18	37	1	1
10–12 years	48	27	3.65 (1.75, 7.62)**	3.57 (1.33, 9.62)*
**Sex of the student**				
Male	19	24	1	1
Female	40	47	2.64 (1.24, 5.65)*	2.71 (0.89, 8.26)
**Family size**				
< =5	50	63	1	1
> 5	14	3	0.17 (0.05, 0.63)*	0.14 (0.02, 0.77)*
**Family education**				
Informal education	26	25	1	^a^
Formal education	38	41	1.12 (0.56, 2.27)	^a^
**Geographic location**				
Lowland	37	39	1	1
Highland	27	27	0.95 (0.47, 1.91)	0.22 (0.06, 0.75)*
**Duration of stay**				
< =1 year	2	3	1	^a^
> 1 year	62	63	0.68 (0.11, 4.19)	^a^
**Family income/month**				
< 500 Eth. Birr	32	36	1	
500–1500 Eth. Birr	28	25	0.79 (0.39, 1.63)	^a^
> 1500 Eth. Birr	4	5	1.11 (0.27, 4.50)	^a^
**Cassava Consumption**				
Never Consume	57	23	1	1
Consume	7	43	15.22 (5.98, 8.74)**	44.82 (11.0, 182.6)**

*P.Value< 0.05 **P.Value< 0.001

^**a**^Not appeared in the final model (not significant) when backward LR method used

^b^Sufficient (UIC > 100 μg/l) ^c^Insufficient (UIC < 100 μg/l)

### Factors associated with goiter

As done for the UIC level, the same analyses were carried out for goiter. According to the result, age and sex of children, family education and monthly income were significantly associated with goiter in the bivariate logistic regression analysis. However, in multivariable logistic regression analyses only age and sex of children and family monthly income were significant and remained the independent predictors of goiter (Table 4).

**Table 4:** Factors associated with goiter at primary school children in Dawro zone, southwest Ethiopia, 2017, (*n* = 652)

Variables with Category	Goiter Status ^**b**^No ^**c**^Yes		Crude OR (95% CI)	Adjusted OR (95% CI)
**Age of the children**				
7–9 years	200	156	1	1
10–12 years	96	200	2.67 (1.94, 3.68)**	2.23 (1.32, 3.77)*
**Sex of the children**				
Male	220	77	1	1
Female	76	279	10.49 (7.29, 15.08)**	9.20 (5.30, 15.97)**
**Family size**				
< =5	190	226	1	^**a**^
> 5	106	130	0.85 (0.75, 1.42)	^**a**^
**Family educational status**				
No formal education	154	231	1	^**a**^
Formal education	142	125	0.59 (0.43, 0.80)**	^**a**^
**Geographic location**				
Lowland	152	174	1	^**a**^
Highland	144	182	1.10 (0.81, 1.50)	^**a**^
**Duration of stay in area**				
< =1 year	6	16	1	^**a**^
> 1 year	290	340	0.44 (0.17, 1.14)	^**a**^
**Family monthly income**				
< 500 Eth. Birr	32	311	1	1
500–1500 Eth. Birr	200	35	0.02 (0.01, 0.03)**	0.02 (0.01, 0.04)**
> 1500 Eth. Birr	64	10	0.02 (0.01, 0.03)**	0.02 (0.01, 0.04)**
**Cassava Consumption**				
Never consume	139	150	1	^**a**^
Consume	157	206	1.22 (0.89, 1.66)	^**a**^

*P.Value< 0.05 **P.Value< 0.001

^**a**^Not appeared in the final model (not significant) when backward LR method used

^b^No = Goiter (neck swelling) not detected during thyroid gland examination

^c^Yes = Visible and/or palpable thyroid swelling was detected during thyroid gland examination

## Discussion

According to WHO/UNICEF/IGN, the median urinary iodine concentration in school children is a good indicator of the iodine status in the general population [32]. In this study the median UIC was 96.12 μg /l. Even though this median UIC level was slightly below normal, more than half, 66 (50.8%) of the children had UIC < 100 μg/l. However; we cannot use this value to categorize iodine status of the study population, since UIC value of an individual child cannot be used to categorize iodine status in any population [2, 31, 32].

The median UIC finding of this study (96.12 μg /l) was better than reports from south Ethiopia, Hawassa town (1.9 μg /l) [24] and Burie and Womberma district of west Gojam in Ethiopia (5 μg/l) [23]. The differences could be related to efforts made by Ethiopian government and other partner’s with regard to salt iodization, distribution, awareness creation of public through trained HEWs which could have increased the demand for iodized salt utilization compared to the periods where other studies were conducted.

Prevalence of goiter is considered as a public health problem if it is equal to or greater than 5% [2] and endemic if it exceeds 10% [32]. In this study setting, the prevalence of goiter was found to be 54.6%, confirming that it is an endemic and severe public health problem. The finding was in line with the reported prevalence of Burie and Womberma district in Ethiopia (54.5%) [23], but higher than the reports from northwest Ethiopia, Metekel zone (39.5%) [26] and Dabat district (29.1%) [11]. It is also higher than the reported prevalence from one of the districts of India (7.75%) [5] and Nigeria (40.2%) [10].

The high goiter prevalence in current study setting could be attributed partly to the mountainous and hilly nature of the area and high rainfall in most seasons of the year which might have resulted in leaching away iodine from top soil for long time. The other reason could be related to the time of study. Those studies conducted in other parts of Ethiopia and cited here [11, 26] were done after the implementation of USI program in the country in 2011. Therefore; the program implementation and enforcement might have contributed to the reduction of goiter prevalence in some accessible areas of the country.

As indicated above, there was discrepancy between median UIC (96.12 μg /l, nearly normal) and goiter prevalence (54.6%, high). The observed difference in this study may reflect the fact that goiter is an indicator of chronic ID. That is, some of the cases currently with normal iodine intake may be the old iodine deficient one who already developed it before but it takes long time for the thyroid enlargement to resolve and they are still living with goiter.

When we assess the iodine content of salt sample at household level, almost two-third (63.5%) contains inadequate iodine. This result shows not the absence of iodized salt in the area rather the insufficiency of iodine in the salt. This could be due to loss of iodine under local conditions in the supply- chain management from time of iodization (production level) to time of distribution at consumer level. A study conducted in Ethiopia by Shawel D. et al. revealed 57% reduction of iodine concentration in the salt sample from the production site to the consumers (household level) [14]. However, the aim of this study was not to evaluate the loss of iodine and we invite further research in this area.

Goitrogenic food like cassava, cabbage, kale and sorghum were consumed with different frequency in current study area (Table 2), but only cassava consumption was significantly associated with UIC level in children. The possible explanation for this could be the presence of cyanogenic glycosides in these food groups other than cassava could be low and within the tolerable ranges. Those children who consume cassava were more likely to have low level of UIC than the non-consumer counter parts (*P* < 0.001). This was in line with similar studies conducted somewhere in Ethiopia [6, 13].

Goiter was more prevalent in female than male students [AOR = 9.20; 95% CI: 5.30, 15.97] and about 2 times higher in children aged 9–12 years as compared to age group 7–9 years [AOR = 2.23; 95% CI: 1.32, 3.77]. Both results were in line with previous reports from Ethiopia (9, 25]. As age increase in school children, the iodine demand increase in both sexes due to physical and physiological changes in the body. Females are more vulnerable than males due to early puberty, inhibitory effect of estrogen on iodine uptake and also its goitrogenic effect by increasing thyroid follicular proliferation [12].

Children of those families who gain higher income (> 500 Eth. Birr) per month were less likely develop goiter as compared to the lower income groups (< 500 Eth. Birr) [AOR = 0.02; 95% CI: 0.01, 0.04]. The odds of developing goiter among children from households with medium to high wealth status were lower as compared to children from poor (low income) households. This finding was supported by the recent studies done in Assosa town, west Ethiopia and Dabat district, northwest Ethiopia [6, 11]. The lower prevalence of goiter in households with higher wealth index could be related to household’s food purchasing power and food security status. Rich family has better access of both iodized salt and variety food. This in turn improves the child’s dietary diversity and prevents him from goiter.

Children with higher age group (10–12 years) were about 3.6 times more likely to have insufficient UIC level than the lower age groups (7–9 years), [AOR = 3.57; 95% CI: 1.33, 9.62]. As age increases the iodine requirement also increased in this age group to support rapid growth (pubertal growth spurt). Moreover; this age is the period of early adolescence where iodine requirement increased due to physiological and hormonal changes. To compensate this requirement the body utilizes more iodine as supported by several studies [9, 17, 23].

Even though, UIC level was significantly associated with students sex in bivariate analysis, it was not significant during multivariable logistic regression analysis. In previous studies UIC level was significantly associated with sex, Ethiopia [25] and Nigeria [18]. The discrepancy could be attributed to the small sub-sample size of UIC (*n* = 130).

Children living in highland area were less likely to have sufficient UIC level than their counter parts in lowland. This could be attributed to the fact that highland area is more mountainous and characterized by high rainfall than the low one. This leads to erosion and leaching away of iodine from top soil. Consequently; crops grown in such area can’t provide sufficient iodine when consumed leading to low level of UIC [2, 13, 20].

There was significant association between cassava consumption and the levels of UIC. Children who consume cassava were more likely to have low level UIC relative to the non-consumer ones [AOR = 44.82, 95% CI, (11.00, 182.61). Here, the wider confidence interval could be a reflection of smaller sub-sample of UIC. Similar result was observed in recent study conducted by Gabriel M, et al., 2014 in Nairobi, Kenya [19].

It is well known that consumption of cassava (including the frequency and duration of consumption) has been shown as a risk factor for goiter development among children [20]. However; in this study there was no association between cassava consumption and goiter. This could be related to the time of introduction of cassava. It is well known that goiter is the long term consequence of iodine deficiency but cassava was introduced to Dawro zone more recently and propagated to lowland kebeles around Omo River bordering Wolaita zone.

### Strength and limitation of the study

Both school and community-based recruitment strategies were used with multiple indicators (UIC, goiter grade, salt iodine content and interview) to measure the prevalence of IDD in the study population. Despite the strength, this study is not free of limitations. One of the limitations was the method used to test the salt samples. We tested salt samples using iodine rapid test kit which shows only the presence or absence of iodine in the salt (qualitative). If Iodometric titration test was done, it would have estimated the iodine content of the salt samples quantitatively. But, we believe this does not affect our finding and conclusions related to the objective of the study, because our primary aim was not to evaluate the iodine content of the salt.

The other limitation was both urine and salt samples were collected from sub-samples, due to resource shortage. However; the sample size of the sub-samples is still fairly sufficient to estimate the prevalence of IDD in the study population. The last limitation to be acknowledged could be the nature of the study design (cross-sectional). In this type of study, it is hard to explain the causal relationship between IDD and its predictors, rather the finding alerts one to conduct further research using more controlled methods to identify causal relationship and to appreciate whether the salt is not iodized at site of production or due to post-production losses at different levels of supply-chain.

## Conclusion

In this study, the median UIC was 96.12 μg /l which is close to the WHO recommended cut-off 100 μg/L. When we observe the UIC values for the individual children, 66 (50.8%) of the children had inadequate iodine intake (UIC < 100 μg/l). However; UIC cannot be used to categorize iodine status in an individual. Therefore, based on a median UIC (96.12 μg /l), the study population is mildly iodine deficient. In addition, there was low concentration of iodine in the salt, high consumption of cassava (goitrogenic) and high prevalence of goiter indicating the presence of severe chronic iodine deficiency and the potential risk in the area. Based on these findings, intensifying efforts in the IDD elimination activities like iodized salt quality control, iodine supplementation for more vulnerable groups and teaching community on techniques of cassava processing (to remove cyanide) for household consumption is highly recommended.

## Data Availability

All data used and/or analyzed during the current study are available from the corresponding author on reasonable request.

## Abbreviations

EDHS: Ethiopian demographic and Health Survey
HEWs: Health extension workers
IDD: Iodine deficiency disorder
IGN: Iodine global network
IQ: Intelligence quotient
PPM: Parts per million
SAC: School age children
SNNPR: Southern Nation Nationalities and People region
UIC: Urinary iodine concentration
UNICEF: United Nations children’s fund
USI: Universal salt iodization
WHO: World health organization
